# A Pleiotropic Role for the Orphan Nuclear Receptor Small Heterodimer Partner in Lipid Homeostasis and Metabolic Pathways

**DOI:** 10.1155/2012/304292

**Published:** 2012-04-22

**Authors:** Gabriella Garruti, Helen H. Wang, Leonilde Bonfrate, Ornella de Bari, David Q.-H. Wang, Piero Portincasa

**Affiliations:** ^1^Section of Endocrinology, Department of Emergency and Organ Transplantations, University of Bari “Aldo Moro” Medical School, Piazza G. Cesare 11, 70124 Bari, Italy; ^2^Division of Gastroenterology and Hepatology, Department of Internal Medicine, Edward Doisy Research Center, Saint Louis University School of Medicine, 1100 S. Grand Boulevard, Room 205, St. Louis, MO 63104, USA; ^3^Department of Biomedical Sciences and Human Oncology, Clinica Medica “A. Murri”, University of Bari Medical School, Piazza G. Cesare 11, 70124 Bari, Italy

## Abstract

Nuclear receptors (NRs) comprise one of the most abundant classes of transcriptional regulators of metabolic diseases and have emerged as promising pharmaceutical targets. Small heterodimer partner (SHP; NR0B2) is a unique orphan NR lacking a DNA-binding domain but contains a putative ligand-binding domain. SHP is a transcriptional regulator affecting multiple key biological functions and metabolic processes including cholesterol, bile acid, and fatty acid metabolism, as well as reproductive biology and glucose-energy homeostasis. About half of all mammalian NRs and several transcriptional coregulators can interact with SHP. The SHP-mediated repression of target transcription factors includes at least three mechanisms including direct interference with the C-terminal activation function 2 (AF2) coactivator domains of NRs, recruitment of corepressors, or direct interaction with the surface of NR/transcription factors. Future research must focus on synthetic ligands acting on SHP as a potential therapeutic target in a series of metabolic abnormalities. Current understanding about the pleiotropic role of SHP is examined in this paper, and principal metabolic aspects connected with SHP function will be also discussed.

## 1. Introduction

Nuclear receptors (NRs) constitute a unique family of ligand-modulated transcription factors. NRs mediate cellular response to small lipophilic endogenous and exogenous ligands [1, 2] and are responsible for sensing a number of hormones, including steroid and thyroid hormones, and act as positive and negative regulators of the expression of specific genes [3–5]. Therefore, NRs play a central role in many aspects of mammalian development, as well as lipid homeostasis, physiology, and metabolism. NRs make up one of the most abundant classes of transcriptional regulators in the body and have emerged as promising pharmaceutical targets.

Classically, NRs consist of several functional domains, that is, a variable N-terminal ligand-independent transactivation domain (which often exhibits a constitutive transcription activation function (AF-1)), a highly conserved DNA-binding domain (DBD) that contains two zinc fingers, a hinge domain (a variable linker region), and a multifunctional C-terminal domain. Furthermore, the C-terminal domain includes the ligand binding (LBD), the dimerization interface, and the ligand-dependent transactivation domain AF-2 [1, 6].

Small heterodimer partner (*SHP*; NR0B2 for nuclear receptor subfamily 0, group B, member 2; MIM number 604630, 601665) is a member of the mammalian NR superfamily, due to the presence of a putative ligand-binding domain (LBD) [7]. SHP functions as a corepressor through heterodimeric interaction with a wide array of nuclear receptors and repressing their transcriptional activity. SHP achieves its goal via several members of the NR superfamily that are able to regulate SHP expression. However, SHP is also a unique and atypical NR because it lacks the classical DNA-binding domain (DBD), generally present in other NRs [8]. The *NR0B *family of NRs consists of 2 orphan receptors: SHP and DAX-1 (dosage-sensitive sex reversal adrenal hypoplasia congenita (AHC) critical region on the X chromosome, gene 1). DAX1 is a gene whose mutation causes the X-linked adrenal hypoplasia congenita [9] and is the only family member that lacks a conventional DBD. DAX-1 (NR0B1) is therefore seen as the closest relative of SHP in the NR superfamily [10–12]. Both SHP and DAX-1 appear to be specific to vertebrates. In this respect, no homologous genes have been found in *Drosophila melanogaster *or *Caenorhabditis elegans* [12]. Whereas SHP is different from other conventional NRs both structurally and functionally, it acts as a ligand-regulated receptor in metabolic pathways [13]. SHP belongs to the orphan subfamily since there is no known ligand for this receptor, except for some retinoid-related molecules [14]. SHP inhibits transcriptional activation by working on several other nuclear receptors, that is, directly modulating the activities of conventional nuclear receptors by acting as an inducible and tissue-specific corepressor [12, 15]. The discovery of SHP dates back to 1996 [10]; since then, this orphan NR has been identified as a key transcriptional regulator of signaling pathways [8, 16] involving fundamental biological functions and metabolic processes. Such processes include cholesterol, bile acid and fatty acid metabolism, glucose and energy homeostasis, and reproductive biology [17]. Experiments performed by fluorescence *in situ *hybridization (FISH) analysis of the human metaphase chromosome have shown that SHP is found at a single locus on chromosome 1 at position 1p36.1 and consists of two exons and a single intron spanning approximately 1.8 kb with 257 amino acids in humans [18]. In mice and rats, SHP resides on chromosomes 4 and 5, respectively, both consisting of 260 amino acids. SHP expression is predominantly observed in the liver [10, 18], but it is also detected at lower levels in other tissues, including the pancreas, spleen, small intestine, colon, gallbladder, kidney, adrenal gland, ovary, lung, brainstem, cerebellum, heart, and thymus (Table 1) [19–21].

**Figure 1:**
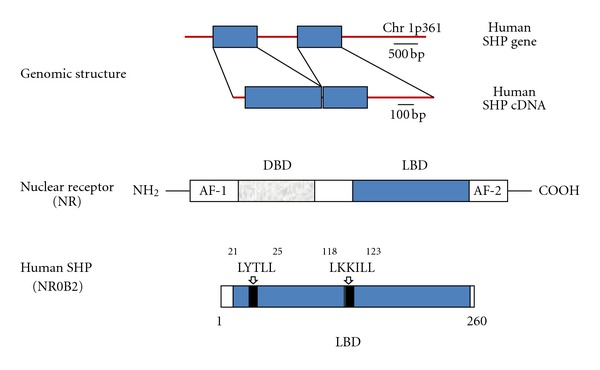
Top: the genomic structure of human SHP. Rectangles represent the two exons with a single intron spanning approximately 1.8 kilobases and located on a single locus on chromosome 1p36.1 [18]. The region 5′ includes *≈*600 nucleotides from the transcription start site and is characterized by promoter activity. Bottom: typical nuclear receptor is compared with the domain structure of human SHP. The canonical structure of NR includes the N-terminal activation function 1 (AF1) domain, DNA-binding domain (DBD), ligand-binding domain (LBD), and C-terminal activation function 2 (AF2) domain. SHP lacks the DBD. Two functional LXXLL-related motifs (also named as NR boxes) are typical of the human SHP structural domains. Such motifs are located in the putative N-terminal helix 1 of the LBD and in the C-terminal region of the helix 5. While active NRs exhibit glutamic acid in AF-2, the SHP AF-2 domain is replaced with aspartic acid. Adapted from Chanda et al. [15] and Shulman and Mangelsdorf [130].

The genomic structure and human SHP domain structure are depicted in Figure 1 [15]. SHP is indeed able to repress the transcriptional activities of its target NRs and transcriptional regulators through two functional Leu-Xaa-Xaa-Leu-Leu- (LXXLL-) like motifs [22–24]. Such motifs appear to be essential for the interaction with the (activation function 2) AF-2 domains of several sets of NRs [22, 23]. The human SHP is enriched by another 12 amino acids [25–36], and this region between helix 6 and 7 is also involved in the repression of the transactivation of NRs [37].

About half of all mammalian NRs and several transcriptional coregulators can interact with SHP [12]. Since SHP lacks DNA-binding domain, it exerts the inhibitory effects through protein-protein interaction [10]. SHP expression seems to follow a circadian rhythm in the liver, involving the CLOCK-BMAL1 pathway and suggesting that some of the regulatory functions of SHP and deriving functions must be temporal [19, 20, 38].

Gene expression of SHP is regulated by several factors including NRs, transcription factors, and a number of additional conditions and substances, as extensively reported in Table 2. Also, the central role of SHP is clear since this NR is able to act as a coregulator for wide range of targets, namely, NRs/transcription factors/transcriptional coregulators and few different molecules, as depicted in Table 3. In general, SHP acts as a repressor of the transcriptional activity of the specific interacting partner (via LBD of the partner and NR boxes of SHP) [12, 39–43]. However, it is also demonstrated that SHP is able to upregulate gene transcription, as in the case of PPAR*α* and PPAR*γ* [44–46] and NF-*κ*B [44].

**Figure 2:**
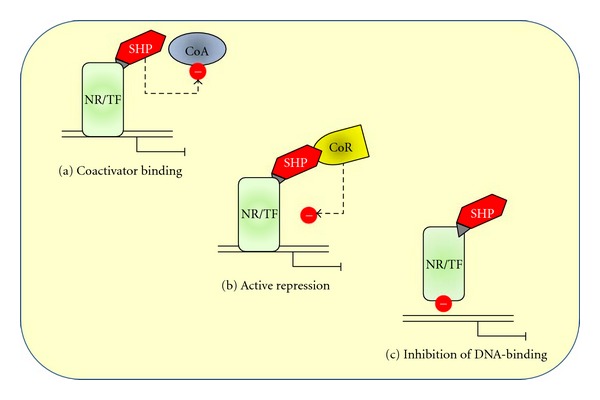
The SHP-mediated repression of target transcription factors occurs by at least three distinct transcriptional repression mechanism: (a) direct interference with the AF-2 coactivator domain of NRs (competition for coactivator binding, leading to the repression of NR-mediated transcriptional activity); (b) recruitment of corepressors, resulting in active repression; (c) direct interaction with the surface of NR or transcription factor, resulting in the blockade of DNA binding and the consequent inhibition of its transcriptional activity. See text for details. The dotted arrows and (-) symbols indicate inhibition. CoA: coactivator; CoR: corepressor; NR: nuclear receptor; SHP: small heterodimer factor; TF: transcription factor. Modified after [12, 15, 131].

Both N-terminal NR interaction domain and C-terminal domain of SHP are important for repression [47, 48]. Overall, the SHP-mediated repression of target transcription factors occurs by at least three distinct transcriptional repression mechanisms (Figure 2).

A first mechanism involves direct interference with the AF-2 coactivator domain of NRs (competition for coactivator binding, leading to the repression of NR-mediated transcriptional activity). This is the case for the inhibition of estrogen receptor *α* (ER*α*) and estrogen receptor *β* (ER*β*) [49].

A second mechanism for the SHP-mediated repression involves the recruitment of corepressors including direct interactions among mammalian homolog of the *Saccharomyces cerevisiae* transcriptional corepressor Sin3p (mSin3A), human Brahma (Brm), SWItch/Sucrose NonFermentable (SWI/SNF) complexes leading to the repression of cholesterol 7*α*-hydroxylase (CYP7A1) [50]).

A third mechanism of inhibition of SHP involves the direct interaction with the surface of NR or transcription factor, resulting in the blockade of DNA binding and the consequent inhibition of its transcriptional activity. This is the case for RAR-RXR heterodimers [10], PXR-RXR binding to DNA by SHP [1], interaction with hepatocyte nuclear factor (HNF4), or Jun family of the activator protein 1 (AP-1) transcription factor complex (JunD) [51, 52].

All three mechanisms might occur sequentially or alternatively according to type of cells and promoters [12].

Clearly, information on factors that increase or decrease SHP expression and that are regulated by SHP is essential for understanding the regulatory effects of this orphan NR. Few years of research have not been enough to identify a true ligand. Interestingly, it is suggested that targeting posttranslational modifications of SHP may be an effective therapeutic strategy. Selected groups of genes could be controlled to cure a vast range of metabolic and SHP-related diseases [53]. Overall, the huge amount of information on SHP function is currently available, making this NR essential in a number of functions involving cholesterol and bile acid metabolism, lipogenesis, glucose metabolism, steroid hormone biosynthesis, xenobiotic homeostasis/metabolism, and cell cycle.

In particular, the ability of SHP in interacting with different metabolic signaling pathways including bile acids and lipid homeostasis, fat mass, adipocytes, and obesity will be reviewed here. 

## 2. Bile Acids and Lipid Homeostasis

The wide ability of SHP to target multiple genes in diverse signaling pathways points to the key role of SHP in various biological processes, including the metabolism of bile salts, glucose, and fatty acids. Both unique structure and functional properties account for the complexity of SHP signaling. Studies suggest that loss of SHP might positively affect cholesterol and bile acid homeostasis in pathophysiologically relevant conditions [54]. Bile acids (BAs) are amphipatic cholesterol metabolites which are synthesized in the liver, secreted into bile, stored in the gallbladder, and secreted postprandially into the duodenum. BAs are synthesized from cholesterol, and this pathway provides the elimination of excess cholesterol in the body [55]. Moreover, BAs should be seen as physiological detergents which, in the small intestine, are essential for the absorption, transport, and distribution of lipophilic molecules, including dietary lipids, steroids, and lipid-soluble vitamins. In the intestine, BAs undergo extensive metabolism by the intestinal microflora. A high efficient system is the enterohepatic circulation of BAs [55, 56], where more than 90–95% of BAs are returned to the liver from the terminal ileum via the portal vein. Thus, the concentration of BAs in serum, liver, and intestine is tightly regulated to prevent damage to enterohepatic tissues due to their strong detergent moiety [57–59]. The major rate-limiting step in biosynthetic pathway of BAs in humans is initiated by cholesterol 7*α*-hydroxylase (CYP7A1), the microsomal P450 liver enzyme, to produce two primary BAs, cholic acid, and chenodeoxycholic acid, essential in the overall balance of cholesterol homeostasis. Sterol 12*α* hydroxylase (CYP8B1) catalyzes the synthesis of cholic acid, a step which determines the cholic acid to CDCA ratio in the bile [60]. Secondary bile acids (deoxycholic acid and lithocholic acid) and tertiary bile acids (ursodeoxycholic acid) in humans are produced following intestinal dehydroxylation of primary bile acids by intestinal bacteria [58, 61].

Regulation of BA biosynthesis is highly coordinated and is mediated by key NRs including the orphan receptor, liver receptor homologue-1 (LRH1; NR5A2), the hepatocyte nuclear factor 4*α* (HNF4*α*), SHP, and the bile acid receptor farnesoid X receptor (FXR; NR1H4). Thus, the activation of FXR initiates a feedback regulatory loop via induction of SHP, which suppresses LRH-1- and HNF4*α*-dependent expression of the two major pathway enzymes cholesterol 7hydroxylase (CYP7A1) and sterol 12 hydroxylase (CYP8B1).

**Figure 3:**
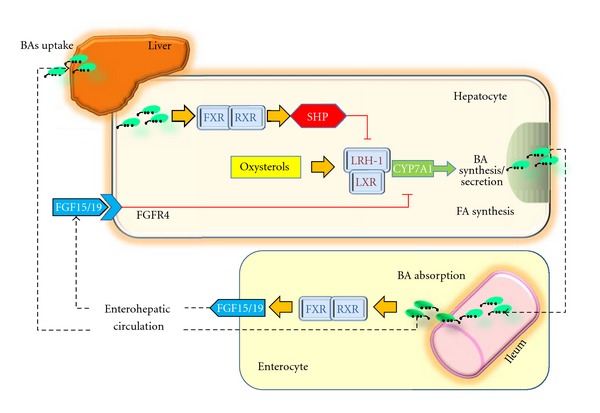
The potential molecular mechanisms of crosstalk between nuclear receptors LXR and FXR–SHP–LRH-1 regulatory cascade in the liver and intestine. Bile acids act as ligands for FXR, which regulates transcription by binding as a heterodimer with RXRs. This step results in increased SHP expression. SHP in turn inhibits LRH-1, preventing the activation of target genes that participate in bile acid and fatty acid synthesis. In the absence of bile acids, LRH-1 acts together with LXR to stimulate bile acid synthesis [64, 65, 132]. The important pathways in the intestine that contribute to modulation of bile acid synthesis are also depicted (see text for details). There is a bile-acid-mediated activation of intestinal FXR and, as a result, the release of FGF15 in the small intestine. The secreted FGF15 by the intestine circulates to the liver, likely through the portal circulation or lymph flow [81] and induces the activation of FGFR4 in the liver. The FGF15/FGFR4 pathway synergizes with SHP *in vivo* to repress CYP7A1 expression [57]. Bas: bile acids; FGF: fibroblast growth factor; FGFR4: FGF receptor; FXR: farnesoid X receptor; LRH-1: liver receptor homologue-1; LXR: liver X receptor; RXR: retinoid X receptors; SHP: short heterodimer partner. Adapted from Ory [66] and Inagaki et al. [57].

The BA feedback regulation primarily occurs since BAs act as transcriptional regulators for the expression of the gene encoding CYP7A1. Both cholic acid and chenodeoxycholic acid function as endogenous ligands for the nuclear bile acid receptor FXR [62]. FXR expression is high in the intestine and liver, the two sites where BAs reach high concentrations to activate FXR. The transcription by FXR includes heterodimerization with retinoid X receptors (RXRs) in the cytoplasm, translocation into the nucleus, and binding to DNA response elements in the regulatory regions of target genes [63]. When the bind of BAs to FXR, SHP transcription is increased [60, 64, 65], this alteration leads to the inhibition of LRH-1 activity or HNF4*α* on the BA response elements (BAREs) of CYP7A1 and CYP8B1 promoters [64, 65]. In this scenario, BA synthesis is downregulated by a precise feedback regulatory mechanism, which represents the major pathway under normal physiological conditions [64–66] (Figure 3). LRH1 is also a well-known activator of *Shp* gene transcription [64, 65], and this step leads to an autoregulatory loop of gene expression by SHP [42]. This step also includes the G protein pathway suppressor 2 (GPS2) interacting with FXR, LRH-1, and HNF4*α* to regulate CYP7A1 and CYP8B1 expression in human hepatocytes [67] (Table 3). A critical role in maintaining cholesterol homeostasis for CYP7A1 has been recently advocated in a model of in Cyp7a1-tg mice [68].

The hepatocyte nuclear factor-1*α* (HNF1*α*), which haploinsufficiency causes the Maturity-onset diabetes of the young type 3 (MODY3), also appears to modulate SHP expression via the FXR pathway. In this respect, HNF1*α* (−/−) mice displayed a defect in bile acid transport, increased bile acid and liver cholesterol synthesis, and impaired HDL metabolism [69].

A role for SHP in mediating the recruitment of mSin3A-Swi/Snf to the CYP7A1 promoter, with chromatin remodeling and gene repression, has been described. In HepG2 cells, Kemper et al. [50] have shown that bile acid treatment resulted in SHP-mediated recruitment of transcriptional coregulators mSin3A and Swi/Snf complex to the promoter, chromatin remodeling, and gene repression (Table 3). This is an additional mechanism involving transformation of nucleosome conformation for the repression by SHP of genes activated by various NRs. In line with such results, increased synthesis and accumulation of BAs occurs in SHP (−/−) mice, due to the loss of SHP repression and consequent derepression of the rate-limiting CYP7A1 and cholesterol 12*α*-hydroxylase (CYP8B1) (the rate-determining enzyme of the alternative but minor BA synthesis pathway) in the biosynthetic pathway [70–72].

Mechanisms independent of the FXR/SHP/LRH pathway might also exist, since BAs feeding to SHP (−/−) mice reduced the levels of CYP7A1 mRNA to similar levels of control mice [70, 71]. Such SHP-independent and alternative pathways include the protein kinase C/Jun N-terminal kinase (PKC/JNK) pathway [73], the FXR/FGFR4 (FGF receptor 4) pathway [57, 74], the cytokine/JNK pathway [75], the pregnane X receptor (PXR) mediated pathway [76], and the JNK/c-Jun signaling pathway [77].

Another study demonstrated, in SHP (−/−) mice on a background of 129 strain, the protection against hypercholesterolemia in three different models: an atherogenic diet, hypothyroidism, and SHP (−/−) mice intercrossed with LDLR (−/−) mice (to generate SHP/LDLR double (−/−) mice in a mixed 129-C57BL/6 background). When fed an atherogenic diet, the latter strain was almost completely resistant to diet-mediated increases in triglyceride, very low-density lipoprotein (VLDL) cholesterol, and low-density lipoprotein (LDL) cholesterol but had an increase in high-density lipoprotein (HDL) cholesterol as compared with LDLR (−/−) mice. Such results point to the protection against dyslipidemia following the inhibition of hepatic SHP expression, although no antagonist ligands have yet been identified for SHP [78]. We have recently examined biliary lipid secretion and cholesterol gallstone formation in male SHP (−/−) and (+/+) mice before and during the feeding of a lithogenic diet for 56 days [79]. Deletion of the *Shp* gene significantly increased hepatic bile salt synthesis, and doubled the increase of biliary bile salt outputs in SHP (−/−) mice than in (+/+) mice. The intestinal bile acid pool size was significantly greater in SHP (−/−) mice than in (+/+) mice. These increased BAs are efficacious ligands of FXR and can stimulate the expression of intestinal fibroblast growth factor 15 (FGF15) in mice through the FXR signaling pathway, which is consistent with the expanded bile acid pool size in SHP (−/−) mice. At 14 days on the lithogenic diet, fasting gallbladder volume was significantly larger in SHP (+/+) mice than in (−/−) mice [80].

Indeed, FGF15/19 (mouse and human orthologs, resp.) is another FXR gene target in the intestine and appears to contribute to the fine tuning of bile acid synthesis in the liver. Thus, a model for FXR-mediated repression of bile acid synthesis should also take into account the bile acid-mediated activation of intestinal FXR and FGF15 in the small intestine (while the FXR-SHP pathway is activated in the liver). According to the most plausible view, FGF15 acts as a hormone to signal between intestine and liver. The secreted FGF15 by the intestine circulates to the liver, likely through the portal circulation or lymph flow [81], and induces the activation of FGFR4 in the liver. As shown in Figure 3, the FGF15/FGFR4 pathway synergizes with SHP *in vivo* to repress CYP7A1 expression [57]. In humans, a similar mechanism should involve the FGF19. Of note, activation of FXR transcription in the intestine protected the liver from cholestasis in mice by inducing FGF15 expression and reducing the hepatic pool of BA. This suggests a potential approach to reverse cholestasis in patients [82]. Hepatic fatty acid homeostasis is also regulated by SHP since regulating these genes involves in fatty acid uptake, synthesis, and export [83–87]. In a study exploring global gene expression profiling combined with chromatin immunoprecipitation assays in transgenic mice constitutively expressing SHP in the liver, overexpression of *SHP *in the liver was associated with the depletion of the hepatic bile acid pool and a concomitant accumulation of triglycerides in the liver [84]. By contrast, fat accumulation induced by a high-cholesterol or high-fat diet is prevented by the deletion of *SHP *[88, 89]. The pleiotropic role of SHP can also be found in the case of nonalcoholic liver steatosis since *OB*/*SHP *double (−/−) mice (a model of severe obesity and insulin resistance) became resistant to liver steatosis and showed improved insulin sensitivity [86].

Another interesting role for SHP emerged after it was found that BAs negatively regulate the human angiotensinogen (ANG) gene. ANG is the precursor of vasoactive octapeptide angiotensin II, and BAs act through the SHP pathway by preventing hepatocyte nuclear factor-4 (HNF4) from binding to the human ANG promoter [90].

## 3. Fat Mass, Adipocytes, and Obesity

SHP appears to play a central role in obesity. Human obesity is considered a polygenic disorder characterized by partly known abnormal molecular mechanisms resulting in increased fat mass, with an imbalance between the energy acquired from nutrients that dissipated as heat (i.e., thermogenesis). In this respect, weight stability requires a balance between calories consumed and calories expended [91]. In adipose tissue depots, two main types of adipocytes exist, that is, brown adipocytes and white adipocytes. In several animal species, some adipose tissue sites mainly include brown adipocytes (BATs) and the other contains mainly white adipocytes (WATs). BAT dissipates chemical energy to produce heat either as a defense against cold [92] or as energy expenditure to compensate food intake [93, 94]. The unusual function of BAT might be better understood by considering that they share a common origin with myocytes [95, 96], and BAT was indeed considered something in between muscle and adipose tissue [95]. BAT is deemed as the major site for sympathetic (adrenergic) mediated adaptive thermogenesis; this pathway involves the uncoupling protein-1 (UCP1). WAT is mainly implicated in the regulation of lipid storage and catabolism but also in the synthesis and secretion of adipokines [97–100]. While the percentage of young men with BAT is high, the activity of BAT is reduced in men who are overweight or obese [101]. Thermogenesis unequivocally exists in both humans and animals, and BAT is the major site of thermogenesis which can be increased by environmental factors (i.e., adaptive thermogenesis). In both human and animal species, dietary composition, chronic cold exposure, and exercise may increase thermogenesis [102]. As far as adipose tissue biology is concerned, SHP seems to play a distinct regulatory function in WAT, as compared with BAT. A number of experiments have focused on animal models of obesity and subtle molecular changes. SHP-deficient mice are protected against high-fat-diet-induced obesity [89].

Peroxisome proliferator-activated receptor (PPAR) *γ* coactivator-1 (PGC-1) family members are multifunctional transcriptional coregulators. PGC-1 acts as a molecular switch in several metabolic pathways. In particular, PGC-1*α* and PGC-1*β* regulate mitochondrial biogenesis, adaptive thermogenesis, fatty acid and glucose metabolism, fiber-type switching in skeletal muscle, peripheral circadian clock, and development of the heart [103]. In particular, SHP functions as a negative regulator of energy production in BAT [89] because SHP is a negative regulator of PGC-1*α* expression in BAT. In turn, PCG-1*α* is a coactivator of uncoupling protein 1 (UCP1) which plays a major role in energy dissipation as heat in multilocular BAT of different animal species and humans [104–106]. Fat-specific (BAT) SHP-overexpressed transgenic mice had increased body weight and adiposity. Energy metabolism, however, was increased, and BAT cold exposure function was enhanced with activation of thermogenic genes and mitochondrial biogenesis (enhanced *β*1-AR gene expression and PGC1*α*). Compared with wild-type mice on a high-fat diet, SHP overexpression was associated with enhanced diet-induced obesity phenotype with weight gain, increased adiposity, and severe glucose intolerance. An additional feature of SHP transgenic mice was a decreased diet-induced adaptive thermogenesis, increased intake of food, and decreased physical activity [107]. This leads to the conclusion that, although expressed at low levels in fat, activation of SHP in adipocytes has a strong effect on weight gain and diet-induced obesity [107]. Moreover, if mechanisms linked to energy metabolism and the development of obesity are considered, SHP has distinct roles in WAT and BAT. As previously mentioned, while *SHP* deletion in obese leptin-deficient mice (*ob/ob*) prevented the development of nonalcoholic fatty liver and improved peripheral insulin sensitivity [86], *SHP* deletion did not overcome the severe obesity caused by leptin deficiency. A significant protective effect from obesity by *SHP* deficiency was likely associated with the low basal level of SHP expressed in fat. Adipogenesis appears to be influenced by SHP: when SHP was overexpressed in 3T3-L1 preadipocytes, cell differentiation was inhibited, as well as the accumulation of neutral lipids within the cells. Thus, SHP may act as a molecular switch governing adipogenesis. In particular, SHP appears to be a potent adipogenic suppressor, and preadipocytes are kept in an undifferentiated state through the inhibition of the adipogenic transcription factors and stimulators [108]. Further studies will address whether the loss of *SHP *function results in inhibition of lipid accumulation in adipocytes, similar to what is observed in hepatocytes. In a future clinical setting, treatment of obesity might also include drugs able to mimic or stimulate the effects of SHP. Mutations in the *Shp* gene have also been reported in patients with lipodystrophy carrying four different polymorphisms [109].

SHP mutations may not be considered a common cause of severe obesity. A number of important clinical studies have examined this issue (Table 4); however, Hung et al. [110] in UK examined the relationships between genetic variation in *SHP* and weight at birth, adiposity, and insulin levels in three different populations (the Genetics of Obesity Study) GOOS, the Avon Longitudinal Study of Parents and Children (ALSPAC), and the Ely studies). In the 329 cases of severe early-onset obesity (GOOS study), two novel and rare missense mutations (R34G and R36G) were identified which might in part contribute to obesity in the probands. Furthermore, two common polymorphisms, namely, G171A (12% of subjects with higher birth weight) and −195CTGAdel (16% of subjects with lower birth weight) were found. In the ALSPAC cohort of 1,079 children, the G171A variant was associated with increased body mass index and waist circumference together with higher insulin secretion 30 minutes after glucose load. Thus, whereas mutations in the *Shp* gene cannot be seen as a common cause of severe human obesity, genetic variation in the *Shp* gene locus may influence birth weight and have effects on body size. The effect might ultimately involve insulin secretion by the negative regulation between SHP and the hepatocyte nuclear factor-4*α* (HFN-4*α*), a transcription factor involved in differentiation and function of pancreatic *β*-cells [110].

A possibility is that decreased *SHP* expression or function results in increased HFN-4*α* activity with a cascade of events, including fetal hyperinsulinemia, and increased birth weight. At a later stage, sustained hyperinsulinemia might be responsible of insulin resistance and obesity of the adult [110].

Mutations in the *Shp* gene were also associated with influence on birth weight, mild obesity, and insulin levels in the study by Nishigori et al. on 274 Japanese subjects [111]. Mutations in several genes encoding transcription factors of the hepatocyte nuclear factor (HNF) cascade are associated with maturity-onset diabetes of the young (MODY). MODY is a monogenic form of early-onset diabetes mellitus (defective insulin secretion with normal body weight), and SHP is deemed as a plausible candidate MODY gene; this is because SHP is able to inhibit the transcriptional activity of the hepatocyte nuclear factor-4*α* (HFN-4*α*), a key member of the MODY regulatory network. Thus, further studies have looked for segregation of *SHP* mutations with MODY in a cohort of Japanese patients with early-onset diabetes. In this context, variants in *SHP* appeared to cosegregate with increased body mass index in families, thus contributing to obesity among Japanese subjects. Also, increased risk of morbidity was observed in another study from Japan, examining patients with type 2 diabetes and *SHP* mutations [112].

Major differences, however, might exist in the prevalence and function of *SHP* variants in different populations. Of note, the results from other Caucasian cohorts did not confirm the association between *SHP* mutation and obesity [113, 114]. Echwald et al. conducted an elegant study on the prevalence of *SHP* variants by single-strand conformational polymorphism and heteroduplex analysis among 750 Danish obese men with early-onset obesity [114]. As control, a cohort of 795 nonobese control subjects was genotyped using PCR-RFLP. Functional analyses of the identified coding region variants were performed in both MIN6-m9 and HepG2 cell lines. Five novel variants were identified (including 3 missense variants (c.100C>G [p.R34G], c.278G>A [p.G93D], and c.415C>A [p.P139H]) and 2 silent variants (c.65C>T [p.Y22Y] and c.339G>A [p.P113P])). The previously reported [111] c.512G>C [p.G171A] common polymorphism was identified; however, the prevalence of functional *SHP* variants associated with obesity was considerably lower among Danish subjects (1 out of 750 obese, none of control subjects), compared to the prevalence observed in Japan by Nishigori et al. [111]. Mitchell at al. [113] investigated SHP variants in 1927 UK subjects according to type 2 diabetes, obesity, and birth weight. Although reporting a raised body mass index among homozygous carriers of the 171A variant (<1%), this polymorphism was unlikely to be associated with all three conditions in Caucasians. Taken together, the above-mentioned studies suggest that the 171A variant might contribute only to subsets of polygenic obesity.

## 4. Other Functions of SHP

The existence of multiple interactions of SHP with NRs, transcription factors and transcriptional cofactors (Tables 2 and 3) points to the pleiotropic and central role of SHP in the body.

SHP has been hypothesized to act in glucose homeostasis via complex pathways involving the inhibition of glucocorticoid receptors (GR) in mammalian cells and the inhibition of PGC-1 gene, a coactivator of NRs important for gluconeogenic gene expression and the PGC-1-regulated phospho(enol)pyruvate carboxykinase (PEPCK) promoter. Such steps underscore a physiologically relevant role for SHP in modulating hepatic glucocorticoid action [22]. Following the bile acid-induced induction, SHP inhibited a number of other pathways, including the HNF4*α*-mediated transactivation of the PEPCK and fructose biphosphate (FBP) promoters, as well as the transactivation of the glucose-6-phosphatase (G6Pase) promoter mediated by Foxo1 [115]. The interaction between SHP inhibitory function and the 3 isoforms (*α*, *β*, and *γ*) of the hepatocyte nuclear factor-3 (HNF4) points to the regulatory role of SHP on gluconeogenesis [51]. A role for SHP in insulin secretion pathway has also been reported. Mutations in hepatocyte nuclear factor 1*α* (HNF-1*α*) is associated with maturity-onset diabetes of the young type 3. This condition depends on impaired insulin secretory response in pancreatic beta cells.

Indeed, loss of HNF-1*α* function in HNF-1*α* (−/−) mice resulted in altered expression of genes involved in glucose-stimulated insulin secretion, but also insulin synthesis, and beta-cell differentiation. Pancreatic islets of HNF-1*α* (−/−) mice showed a distinctive reduction of SHP expression and a downregulation of the HNF4*α* gene expression. Since SHP appears to repress its own transcriptional activation following heterodimerization with HNF4*α*, a feedback autoregulatory loop between SHP and HNF4*α* has been hypothesized [116]. Also, SHP likely functions as a negative regulator of pancreatic islet insulin secretion. SHP (−/−) mice were characterized by hypoinsulinemia, increased glucose-dependent response of islets, increased peripheral insulin sensitivity, and increased glycogen stores [117]. The role played by SHP in the regulation of hepatic gluconeogenesis has also emerged in a number of additional experiments. For example, the liver of SHP (−/−) mice showed increased glycogen stores [117], while hepatic *Shp* gene expression (induced by the antidiabetic biguanide drug metformin) was associated with inhibition of hepatic gluconeogenesis. Induction of SHP was achieved via AMP-activated protein kinase (AMPK) and associated with downregulation of essential gluconeogenic enzyme genes, that is, phosphoenolpyruvate carboxykinase (PEPCK), glucose-6-phosphatase (G6Pase) [118], and fructose-1,6-bisphosphatase (FBP1) [119].

PGC-1 gene is a coactivator of NRs, and this step is relevant for gluconeogenic gene expression. Yamagata et al. [119] showed that bile acid (chenodeoxycholic acid) was able to induce the downregulation of PGC-1 gene, and this mechanism involved forkhead transcription factors (Foxo1, Foxo3a, Foxo4) via a SHP-dependent manner.

Drug metabolism and detoxification might be regulated by SHP. This is also the case for excess BAs: the pregnane X receptor (PXR) induces CYP3A and inhibits CYP7*α*, both involved in biochemical pathways leading to the conversion of cholesterol into primary BAs, whereas CYP3A is also involved in the detoxification of toxic secondary bile acid derivatives. SHP acts as a potent repressor of PXR transactivation, and this finding suggests that PXR can act on both bile acid synthesis and elimination detoxification [1]. Additional mechanisms involved in the SHP-dependent control of pathways of drug metabolism have been identified. The expression of genes involved with the metabolism of xenobiotics might be regulated by SHP in the spleen acting on (aryl hydrocarbon receptor (AHR)/AHR nuclear translocator (ARNT)) AHR/ARNT heterodimers which, in turn, bind to xenobiotic response elements (XREs) at the level of specific DNA sequences [41]. A number of genes involved in hormone and drug metabolism would be expressed (i.e., UGT16, ALDH3, CYP1A1, CYP1A2, CYP1B1, etc.). SHP also appears to downregulate the constitutive-androstane-receptor- (CAR-) mediated CYP2B1 gene expression, induced by phenobarbital to form the CAR/RXR heterodimer which, in turn, binds to 2 DR-4 sites to form the phenobarbital responsive unit in the CYP2B gene [120] (Table 3). One role of SHP in steroidogenesis has been identified in the testes with influence on testosterone synthesis and germ cell differentiation [121] and in the intestine for glucocorticoid synthesis [122].

A role for SHP in cell proliferation and apoptosis signaling is emerging. Depending on the cell type, SHP seems to have both inhibitory and stimulatory effects on apoptosis. However, the manipulation of SHP through the synthetic ligands adamantyl-substituted retinoid-related (ARR) compounds 6-[3-(1-adamantyl)-4-hydroxyphenyl]-2-naphthalenecarboxylic acid (CD437/AHPN) and 4-[3-(1-adamantyl)-4-hydroxyphenyl]-3-chlorocinnamic acid (3-Cl-AHPC) induces apoptosis of a number of malignant cells (i.e., leukemia and breast carcinoma) both *in vitro* and *in vivo* [123, 124]. The complex mechanism implies binding of ARR and 3-Cl-AHPC to SHP with formation of a corepressor complex containing Sin3A and nuclear receptor corepressor (*N*-CoR) which activate local control of mitochondrial function and apoptosis, with a limiting function on tumorigenesis [17, 123] (Table 3). SHP appears to be also involved in DNA methylation and acting as a tumor suppressor, at least in the human and mouse livers [125–127]. Whether manipulation of SHP will be helpful in the treatment of hepatic and other gastrointestinal cancers is still a matter of research. The recent finding that SHP negatively regulates TLR signaling to NF-*κ*B has raised the interest for the role of SHP in mechanisms governing innate immunity. SHP appears to negatively regulate the expression of genes encoding inflammatory molecules. Of note, direct binding of NF-*κ*B seems to occur in resting cells, while binding of SHP to TRAF6 occurs in LPS-stimulated cells [128, 129].

## 5. Conclusions and Perspectives

**Figure 4:**
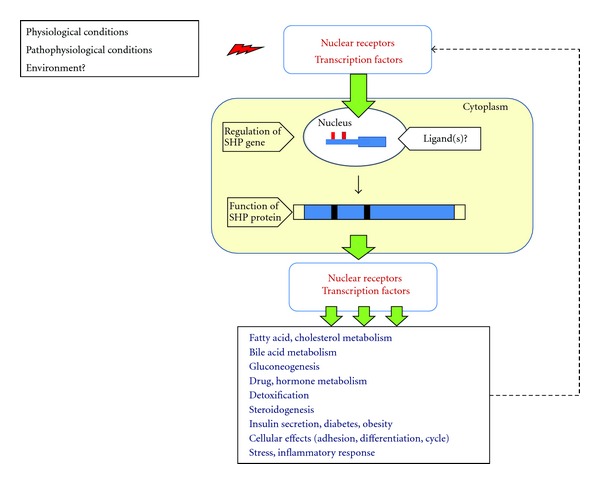
Schematic diagram of the function and gene regulation of SHP. Different conditions will lead to activation of nuclear receptors and/or transcription factors able to regulate *Shp* gene expression in the nucleus and protein synthesis in the cytoplasm. The protein acts as a transcriptional corepressor of a number of other nuclear receptors and transcription factors involved in a wide series of regulatory pathways. The potential role of a feedback mechanism and of ligand(s) is hypothesized.

A remarkable number of metabolic functions in the body appear to be regulated by the orphan unique NR, small heterodimer partner SHP, which targets a complex set of genes in multiple pathways as a transcriptional corepressor (Figure 4). Pathways include fatty acid metabolism, glucose homeostasis, and drug-hormone detoxification. When looking at complex mechanisms leading to some important *lipidopathies, *that is, obesity and liver steatosis, enlightening data about the regulatory function of SHP are provided by studies using *Shp*-deleted and *Shp*-overexpressed animal models. Most likely, a condition of *Shp *deficiency might counteract lipid accumulation and improve plasma lipoprotein profiles. Further studies are urgently needed to confirm that such an important metabolic regulatory mechanism of SHP is true and has high translational value. To date, however, no synthetic antagonists or agonists for SHP are available, and one should keep in mind that rather divergent and somewhat elusive data have been observed regarding the loss of SHP function in humans and rodents. Thus, careful examination of subtle SHP intrinsic functions is essential to dissect potential modulatory pathways of SHP for a variety of metabolic abnormalities but also in tumorigenesis. Moreover, identifying specific endogenous ligands and synthetic agonists of SHP will pave the way to for therapeutic intervention. The effect of synthetic ligands on SHP modulation in hepatocytes and adipocytes, for example, might represent therapeutic tools for the treatment of constituents of the metabolic syndrome, namely, hypercholesterolemia, overweight obesity, and liver steatosis.

## Figures and Tables

**Table 1:** Small heterodimer partner (SHP) expression [10, 18–21].

LIVER (greater)*	
Spleen*	
Pancreas*	
Central nervous system (brainstem and cerebellum)	
Adrenal gland*	
Intestine (duodenum*, jejunum*, ileum*, and colon)	
Gallbladder, stomach*, kidney*, ovary, lung, prostate, testis, uterus, heart*, thymus, and epididymis	

All organs in the mouse. Astericks indicate SHP expression in humans [18, 133].

**Table 2:** Regulators of the Shp gene promoter [12, 39–43].

(1) *Nuclear receptors *	

Protein	Model(s)/putative function

ER*α*	Uterus, pituitary, kidney, and adrenal gland, HepG2 cell lines/biological effects of estrogens, LDL/HDL metabolism [134].

ERR*α*, *β*, *γ*	SHP promoter is activated by the ERR*γ*, while SHP inhibits ERR*γ* transactivation (autoregulatory loop). SHP and ERR*γ* coexpressed in several tissues (e.g., pancreas, kidney, and heart). Role in some forms of moderate obesity? SHP also physically interacts with ERR *α* and *β* isoforms (yeast two-hybrid and biochemical assays) [133].

FXR	Downregulation of CYP7A1-mediated bile acid biosynthesis by the FXR/SHP/LRH-1 cascade in the liver [64].

LXR*α*	Direct regulation of SHP and repression of CYP7A1-mediated bile acid biosynthesis (in humans not in rodents). Effect on cholesterol homeostasis [135].

LRH-1	Liver/formation of heterodimeric SHP/LRH-1 complex > inactivation of LRH-1 > SHP repression (autoregulatory negative feedback) [64, 65, 136]. Also involved in the CLOCK-BMAL1 circadian activation of SHP [38].

PPAR*γ*	Liver/PPAR*γ* decreases gluconeogenic gene expression by the PPAR*γ*/RXR*α* heterodimer binding to the PPRE in the human SHP promoter. A mechanism explaining the SHP-mediated acute antigluconeogenic effects of PPAR*γ* [137].

SF-1	At least five binding sites for SF-1 detected in the promoter region of SHP. Rat testis and adrenal glands, human fetal adrenal gland [136].

(2) *Transcription factors *	

Protein	Model(s)/putative function

CLOCK-BMAL1	Liver/SHP displays a circadian expression pattern involving CLOCK-BMAL1 (core circadian clock component). Regulation of SHP promoter together with LRH-1 and SHP. Relevance for circadian liver function? [38].

E2A proteins (E47, E12, E2/5)	HepG2, HeLa, and CV-1 cells/bHLH transcription factors, the E2A proteins activate human (not mouse) hSHP promoter. E47 and SF-1 stimulate cooperatively SHP promoter. The Id protein inhibits E47 binding to hSHP promoter. A role for tissue-specific gene regulation, B-cell differentiation, tumor suppression? [138].

HNF-1*α*	Liver/modulation of bile acid and liver cholesterol synthesis via the FXR/SHP/LRH-1 complex and effect on CYP7A1 [69].

HNF4*α*	Pancreatic *β*-cells/decreased expression of SHP may be indirectly mediated by a downregulation of HNF4*α*. SHP can repress its own transcriptional activation by inhibiting HNF4 *α* function (feedback autoregulatory loop) and, indirectly (via HNF4 *α*), HNF1*α* function. Relevance for pancreatic islet differentiation, insulin secretion, synthesis [116].

JNK/c-Jun/AP-1	Primary rat hepatocytes/bile acid downregulation of CYP7A1-dependent bile acid biosynthesis via the JNK/cJun/AP1 pathway. SHP promoter is a direct target of activated c-Jun binding to AP-1 element [139]. Also, in HL-60 leukemia cells, c-Jun increases the transcriptional activation of the SHP promoter to activate the expression of Shp genes associated with the cascade regulation of monocytic differentiation [140].

SMILE	HEK-293T, HepG2, MCF-7, T47D, MDA-MB-435, HeLa, PC-3, C2C12, NIH 3T3, K28, Y-1, and TM4 cell lines/SMILE isoforms (SMILE-L and SMILE-S) regulate the SHP-driven inhibition of ERs transactivation in a cell-type-specific manner [25, 26, 39].

SREBP-1	Liver/effect on human (not mouse) SHP promoter. Cholesterol and bile acid homeostasis, fatty acid synthesis [27].

USF-1	HepG2, H4IIE, and AML12 cells/HGF activates AMPK signaling pathway in hepatocytes, E-box-binding transcription factor USF-1, and binding to the Shp gene promoter. SHP induction of gene expression leads to inhibition of hepatic gluconeogenesis due to SHP-repressed transcription factor HNF4*α* [28].

(3) *Transcriptional coregulators *	

Protein	Model(s)/putative function

RNF31	NCI-H295R (H295R) adrenocortical carcinoma cell line, COS-7 and HeLa cells/RNF31 interacts with SHP, stabilizes DAX-1, and is required for DAX-1-mediated repression of transcription. Relevant as coregulator of steroidogenic pathways [43].

SRC-1	Murine macrophage cell line RAW 264.7, HeLa, and CV-1 cells/SHP interacts negatively with SRC-1 (a transcription coactivator of nuclear receptors and other transcription factors including NF-*κ*B). See also oxLDL in this table [44].

(4) *Other SHP inducers *	

Factor	Model(s)/putative function

Bile acids (final intermediates)	Experiments in HepG2 cells/treatment with chenodeoxycholic acid and late intermediates in the classic pathway of bile acid synthesis: 26-OH-THC (5*β*-cholestane-3*α*,7*α*,12*α*,26-tetrol), THCA (3*α*,7*α*,12*α*-trihydroxy-5 *β*-cholestanoic acid), 26-OHDHC (5*β*-cholestane-3*α*,7*α*,26-triol), DHCA (3*α*,7*α*-dihydroxy-5*β*-cholestanoic acid) resulted in 2.4-6.5-fold increase in SHP mRNA expression [132]. Confirmed by Ourlin et al. with the two FXR ligands chenodeoxycholic acid and cholic acid [1].

Guggulsterone (plant sterol)	Active extract from Commiphora Mukul. FXR antagonist. In Fisher rats, guggulsterone increased transcription of bile salt export pump (BSEP) mRNA and SHP expression [29].

GW4064 (ligand)	Synthetic FXR-selective agonist [29]. In primary cultured human hepatocytes, GW4064 treatment was associated with a marked induction of *SHP* (*≈*70-fold) and complete suppression of *CYP7A*1 [64, 65]. In HepG2 cells, GW4064 (1uM) induced a 3.9-fold increase in SHP mRNA expression. Confirmed by [30].

Interleukins (various)	IL-1Ra (−/−) mice/high cytokine levels in IL-1Ra (−/−) mice reduce mRNA expression of CYP7A1 with concurrent upregulation of SHP mRNA expression [31]. SHP significantly expressed in IFN-*γ*/CH11-resistant HepG2 cells [32].

PGC-1*α* (gene expression inducer)	COS-7 cell lines/PGC-1*α* mediates the ligand-dependent activation of FXR and transcription of Shp gene. Relevance in mitochondrial oxidative metabolism in brown fat, skeletal muscle, and liver gluconeogenesis [33].

PMRT1 (group of protein arginine methyltransferases)	Hepatic cell lines/PRMT1 functions as FXR coactivator and has a role in chromatin remodeling. PRMT1 induces BSEP and SHP and downregulation of NTCP and CYP7A1 (targets of SHP) [30].

Procyanidins (polyphenols)	Grape seed procyanidin extract is given orally in male Wistar rats. Increase of liver mRNA levels of small heterodimer partner (SHP) (2.4-fold), cholesterol 7*α*-hydroxylase (CYP7A1), and cholesterol biosynthetic enzymes with improved lipidogenic profile and atherosclerotic risk [34].

(5) *Factors/conditions associated with SHP repression *	

*β* Klotho (type I membrane protein)	In *β*Klotho (−/−) mice: enhanced bile acid synthesis with attenuation of bile acid-mediated induction of *Shp*. *β*Klotho involved in CYP7A1 selective regulation [35].

IL-1*β* (interleukin)	SHP downregulation [36].

oxLDL (oxidized low density lipoprotein)	Murine macrophage cell line RAW 264.7, HeLa, and CV-1 cells/oxLDL decreased SHP expression. SHP transcription coactivator of NF-*κ*B which became progressively inert in oxLDL-treated RAW 264.7 cells (see also Table 3). Relevance for differentiation mechanism of resting macrophage cells into foam cells and resulting atherogenesis [44].

AP-1: adaptor protein-1; bHLH: basic helix-loop-helix; DAX1: dosage-sensitive sex reversal adrenal hypoplasia congenita critical region on the X chromosome, gene 1; E2A: E2A2 gene products belonging to the basic helix-loop-helix (bHLH) family of transcriptor factors; ER*α*: estrogen receptor*α*; ERR*γ*: estrogen receptor-related receptor-*γ*; FXR: farnesoid X receptor; HGF: Hepatocyte growth factor; HNF-1*α*: hepatocyte nuclear factor-1*α*; HNF4*α*: hepatocyte nuclear factor-4*α*; Id: inhibitor of differentiation; IL-1Ra (−/−): interleukin-1 receptor antagonist; JNK: Jun N-terminal kinase; LRH-1: liver receptor homologue-1; LXR*α*: liver X receptor*α*; NF*κ*B: nuclear factor-*κ*B; NR: nuclear receptor; NTCP: Na^+^-taurocholate cotransport peptide; oxLDL: oxidized low-density lipoprotein; PGC-1: PPAR*γ* (peroxisome-proliferator-activated receptor *γ*) coactivator-1*α*; PMRT1: protein arginine methyltransferase type 1; PPRE: PPAR response element; RNF31: member of the ring-between-ring (RBR) family of E3 ubiquitin ligases; RXR *α*: retinoid X receptor; SF-1: steroidogenic factor-1; SHP: small (short) heterodimer partner; hSHP: human small (short) heterodimer partner; SMILE: SHP-interacting leucine zipper protein; SRC-1: steroid receptor coactivator-1; SREBP-1: sterol regulatory element binding protein-1; USF-1: upstream stimulatory factor-1.

**Table 3:** SHP targets [12, 39–43].

(1) *Nuclear receptors *	

Protein	Model(s)/putative function

AR	The AR/SHP interaction leads to >95% inhibition of AR via the LXXLL motifs. Mechanisms involve inhibition of AR ligand-binding domain and AR N-terminal domain-dependent transactivation and competing with AR coactivators [23].

CAR, RAR, TR	HepG2 and JEG-3 cells/early evidence that SHP interacts with several receptor superfamily members and inhibits transactivation. CAR is an NR-inducing CYP2 and CYP3 genes involved in the metabolism of xenobiotics [10, 24].

DAX-1	Human embryonic kidney 293 cells/beside individual homodimerization of DAX1 and SHP, this is the first evidence of DAX1-SHP heterodimerization in the nucleus of mammalian cells. Involvement of the LXXLL motifs and AF-2 domain of DAX1 in this interaction. Distinct functions for SHP (different from transcriptional repressor) are anticipated [141, 142].

ER	293 human embryo kidney cells, Cos7 kidney cells/direct inhibitory binding of SHP to ERs via LXXLL-related motifs to the AF-2 domain [21]. RL95-2 human endometrial carcinoma cells/SHP inhibits the agonist activity of 4-hydroxytamoxifen displaying a potent inhibitory effect for Er*α* > ER*β*. Direct interaction of SHP with ER and inhibition of ER transcriptional activity [143]. Prevention of tamoxifen-induced estrogen agonistic effects and neoplastic changes in the endometrium in women with breast cancer taking tamoxifen?

ERR*γ*	HeLa (human cervical carcinoma), CV-1 (green monkey kidney), and HEK 293 (human embryonic kidney) cell lines/SHP inhibits ERR*γ* transactivation by physical interaction with the 3 members of the ERR subfamily. Interaction is dependent on N-terminal receptor interaction domain of SHP and AF-2 surface of ERR*γ*. Part of the autoregulatory mechanism of gene expression going through ERR*γ*/SHP/ERR*γ*. A potential role in some forms of moderate human obesity during SHP mutations [133].

GR	293 human embryo kidney cells and COS-7 monkey kidney/SHP inhibits the transcriptional activity of GR via the LXXLL motif. Physiological role of SHP in glucocorticoid signaling and gluconeogenesis [22]. See also HNF4 [90] and Foxo1 [115].

HNF4	Human ANG transgenic mice and HepG2 cells treated with bile acids/evidence that bile acids negatively regulate the human ANG gene through the FXR/SHP-mediated process (inhibition of the binding of HNF4 to the ANG promoter) [90]. Mechanisms: SHP binds the AF-2 region and the N-terminal region of HNF4 and inhibits the binding of HNF4 to DNA. Also, modulation of HNF4 activity by SHP has important metabolic effects and interacts with the pathway of gluconeogenesis [47] (see text and Foxo1) [115].

LRH-1	HepG2 cells/SHP interacts directly with the orphan receptor LRH-1 (AF-2 surface) and competes with other coactivators, leading to repression of LRH-1 transcriptional activity [48]. Demonstration that repression of CYP7A1 and bile acid synthesis requires coordinate interaction/transcription of FXR/LRH-1/SHP autoregulatory cascade, essential for maintenance of bile acid-induced negative feedback, and therefore hepatic cholesterol metabolism [65] (see also Figure 2).

LXR*α*	*In vitro *experiments and *in vivo *human colon Caco-2 cells/SHP directly inhibits the transcriptional activity of LXR*α* via the AF-2 domain. Relevance for direct downregulation of specific LXR target genes (controlling CYP7A1, ABCA1, ABCG1, ABCG5, ABCG8, CETP, ApoE, SREBP-1c) and therefore cholesterol-bile acid homeostasis [144].

Nur77 (NGFI-B)	HepG2 cells/Nur77 plays a key role in apoptosis of many cell types and cancer cells. Evidence that SHP functions to repress the transcriptional function of Nur77 (binding coactivator CBP, see elsewhere in this table). SHP plays a protective role in the Nur77-mediated apoptosis in liver. Mutations in SHP: a role also for affect initiation and progression of inflammatory liver diseases such as alcoholic hepatitis and hepatic viral infections? [32].

PPAR*α*	*In vitro *binding assays and *in vivo *experiments/the promoter regions of the genes encoding the first two enzymes of the peroxisomal beta-oxidation pathway (AOx, HD), contain transcriptional regulatory sequences (PPRE) bound by the PPAR*α*/RXR*α* heterodimeric complex. SHP-inhibited transcription by PPAR*α*/RXR*α* heterodimers from the AOx-PPRE. SHP potentiated transcription by PPAR*α*/RXR*α* heterodimers from the HD-PPRE (evidence of SHP-dependent upregulation PPAR*α*-mediated gene transcription) [46].

PPAR*γ*	*In vitro *experiments, COS-7 cells/*Shp* gene expressed also in adipose tissue. SHP induces PPAR activation via C terminus (direct binding to the DBD/hinge region of PPAR*γ*) and inhibition of the repressor activity of NCoR. SHP may act as an endogenous enhancer of PPAR*γ* by competing with NCoR [45]. Mutant SHP proteins display less enhancing activity for PPARy compared with wild-type SHP, and a human model leading to mild obesity and insulin resistance has been described in Japanese during naturally occurring mutations [111] (see also text and Table 3).

PXR	*In vitro *experiments, human hepatocytes, mouse model on cholic acid-supplemented diet/SHP act as potent repressor of PXR transactivation. Upon sensing xenobiotics and bile acid precursors, PXR controls CYP3A gene induction and inhibits CYP7*α*, acting on both bile acid synthesis and catabolism. PXR function might be also inhibited in the presence of cholic acid, chenodeoxycholic acid-dependent SHP upregulation [1].

RXR	HepG2 cells/demonstration that SHP acts as a transcriptional repressor for RXR. Full inhibition by SHP requires its direct repressor activity [47].

SHP	Human embryonic kidney 293 cells/LXXLL motifs and AF-2 domain are involved in SHP homodimerization in the nucleus (similarly to DAX1-SHP heterodimerization). NR0B family members use similar mechanisms for homodimerization as well as heterodimerization. Distinct functions for SHP (different from transcriptional repressor) are anticipated [141, 142].

(2) *Transcription factors *	

Protein	Model(s)/putative function

ARNT	RL95-2 human endometrial carcinoma cells/TCDD binds to AHR (a member of bHLH-PAS family of transcription factors). Studies on physical and functional interaction of SHP with the ligand AHR/ARNT heterodimer showed that SHP inhibits the transcriptional activity of ARNT (not AHR) *in vitro*. Consequent inhibition of binding of AHR/ARNT to XREs. [41]. Relevance for expression of several genes involved in drug and hormone metabolism [145].

BETA2/NeuroD	293T, COS-7, CV-1 cells/BETA2/NeuroD is a member of tissue-specific class B bHLH proteins and cats as a positive regulator of insulin gene expression [146] and neuronal differentiation [147]. SHP physically interacts and inhibits helix-loop-helix transcription factor BETA2/NeuroD transactivation of an E-box reporter in mouse pancreas islets. The inhibitory effect of SHP requires its C-terminal repression domain, interference with coactivator p300 for binding to BETA2/NeuroD, and direct transcriptional repression function. Relevance for development of the nervous system and the maintenance and formation of pancreatic and enteroendocrine cells [148].

C/EBP*α*	HepG2 hepatoma cells/SHP interacts directly with C/EBP*α* and represses C/EBP*α*-driven PEPCK gene transcription. Overall, a role for SHP in regulation of hepatic gluconeogenes is driven by C/EBP*α* activation in the liver [149].

Foxo1	C57BL/6J mice and HepG2 and HEK293T cells/treatment with chenodeoxycholic acid was associated with FXR-dependent SHP induction, downregulation of gluconeogenic gene expression (G6Pase, PEPCK, FBP1), interaction of the forkhead transcription factor Foxo1 with SHP, and repression of Foxo1-mediated G6Pase transcription (competition with CBP). A similar mechanism is postulated for SHP-driven HNF-4 repression of PEPCK, FBP1 transcription. A mechanism by which bile acids metabolism is linked to gluconeogenic gene expression via an SHP-dependent regulatory pathway [115].

HNF3 (Foxa)	HepG2, 293T, NIH3T3, and HeLa cells, primary hepatocytes/SHP physically interacts and inhibits the transcriptional activity of the forkhead transcription factor HNF3 (isoforms *α*, *β*, *γ*). Relevance for SHP-driven regulation of gluconeogenic genes encoding G6Pase, PEPCK, and bile acid synthesis (CYP7A1), via inhibition of DNA-binding of HNF3 [51].

Jun D	Two rat models of liver fibrosis and Hepatic Stellate cells (HSC)/promoting the ligand-induced FXR-SHP cascade (by the FXR ligand 6-EDCA, in rat models) and overexpressing SHP in HSC prevented fibrogenic changes in the liver. SHP binds JunD and inhibits DNA binding of adaptor protein (AP)-1 induced by thrombin. FXR ligands as therapeutic agents to treat liver fibrosis? [52].

NF-*κ*B	Murine macrophage cell line RAW 264.7/SHP acts as a positive transcription coactivator of NF-*κ*B and essential for NF-*κ*B transactivation by palmitoyl lysophosphatidylcholine (one of the oxLDL constituents). Relevance for differentiation mechanism of resting macrophage cells into foam cells and resulting atherogenesis (see also [44]).

Smad	HepG2, CV-1, and HeLa cells/SHP represses Smad3-induced transcription by competing for the coactivator p300. SHP therefore represses TGF-*β*-induced gene expression. Relevance for TGF-*β*-dependent regulation of cell growth, apoptosis, carcinogenesis, and regeneration following liver injury [40]. SHP-Smad3 interaction similar to SHP-BETA2/NeuroD [148].

TRAF6, p65	Macrophages/a novel function of SHP in innate immunity involving Toll-like receptors (TLRs). SHP negatively regulates TLR signaling to NF-*κ*B. Likely, SHP negatively regulates immune responses initiated by various pathogen-recognition receptors by forming a complex with TRAF6 and effect on TRAF6 ubiquitination. In the cytosol of LPS-stimulated cells. SHP also acts as specific transrepressor of the transcription factor p65 (part of the p50/p65 heterodimer found in NF-*κ*B). An additional role for SHP in sepsis and inflammatory disease? [128, 129].

(3) *Transcriptional coregulators *	

Protein	Model(s)/putative function

Brm, BAF155, BAF47, mSin3A, Swi/Snf	HepG2 cells/The *CYP7A1 *gene was used as a model system. SHP has direct interaction with corepressors at the level of native chromatin. SHP directly interacted and mediated the recruitment of mSin3A-Swi/Snf-Brm chromatin remodelling complex to the *CYP7A1 *promoter (TATA and BARE II region of the promoter). Also, the mSinA3/HDAC1 corepressor complex is inhibiting transcription by histone deacetylation. SHP also interacted with known proteins belonging to the Swi/Snf complex (BAF155, BAF47). This mechanism explains the complex and subtle SHP-driven inhibition of hepatic bile acid synthesis [50].

CBP	HepG2 cells, CV-1 cells/SHP binds coactivator CBP and competes with Nur77. The mechanism explains the repression of the transcriptional function of Nur77, which is fundamental in apoptosis in the liver [32].

EID-1	Cos-7 cells/SHP specifically interacts with EID-1 providing inhibitory mechanisms. EID-1 (a non-HDAC cofactor) acts as inhibitor of the coregulator complex EID1–p300–CBP. Results clarify essential repression mechanisms of SHP involving coinhibitory factors (upstream targets) distinct from NRs corepressor [12, 150].

G9a, HDAC-1	Caco-2, HepG2, HeLa, Cos-1 cells/SHP localized exclusively in nuclease-sensitive euchromatin regions. SHP can functionally interact with HDAC-1 (HDAC of class I) and the euchromatic histone 3 methylase G9a, and the unmodified K9-methylated histone 3 [151]. Additional data on mechanisms involved SHP-driven repressive activity, involving also target genes regulated by G9a and SHP-mediated inhibition of hepatic bile acid synthesis via coordinated chromatin modification at target genes [152].

GPS2	Cos-7, HepG2, Huh7 cells/SHP negatively interacts with GPS2 (a stoichiometric subunit of the NR corepressor, N-Cor) complex, involved in bile acid synthesis and differential coregulation of CYP7A1 and CYP8B1 expression [67].

SIRT1	HepG2, HEK293T (293T), and HeLa cells/SIRT1 is a HDAC of class III. SHP recruits SIRT1 (activating deacetylase activity of SIRT1) to repress LRH1 transcriptional activity as well as inhibition LRH1 target gene promoter activity and mRNA levels. A novel mechanism is described for SHP repressive action and control of bile acid homeostasis. SIRT1 in working concertedly with NRs and affecting chromatin remodeling in target gene promoters [42].

SMRT/NcoR	Hepatoma cell lines/studies on the role of SHP in CAR-mediated transactivation of the CYP2B gene. SHP might interact with subunits of functionally distinct coregulator complexes, including HDAC3-N-CoR-SMRT [24, 120].

(4) *Others *	

Factor	Model(s)/putative function

miRNA-206	SHP^−/−^ mice/SHP as an important transcriptional activator of miRNA-206 gene expression via a cascade dual inhibitory mechanism involving AP1 but also YY1 and ERR*γ*. Relevance for multiple steps involving cellular development, proliferation, and differentiation [153].

RNA Pol II	Caco-2 cells/within the pathway of SHP-LXR interaction, it is shown that SHP can interact *in vitro* with RNA polymerase II but not with TFIID and TFIIE transcription initiation factor II D (TFIID), general transcription factor II E (TFIIE) (components of the basal transcription machinery). A further mechanism by which SHP could inhibit both basal and induced transactivation [144].

ABCA1, ABCG1, ABCG5, and ABCG8: ATP-binding cassette transporters; AP1: transcription factor activator protein 1; AHR: aryl hydrocarbon receptor (AHR); ARNT: aryl hydrocarbon receptor (AHR)/AHR nuclear translocator protein; ANG: angiotensin; AOx, acyl-CoA oxidase; ApoE: apolipoprotein E; bHLH-PAS: basic helix–loop–helix–PAS; AR: androgen receptor; BAFs: Brm- or Brg-1-associated factors; BARE: bile acid response element; Brm: human Brahma; CAR: constitutive androstane receptor; CBP: CREB-binding protein; C/EBP*α*: CCAAT/enhancer-binding protein *α*; CETP: cholesteryl ester transfer protein; CREB: coactivator cAMP-response element-binding protein; CYP7A1: cholesterol-7-*α*-hydroxylase; DAX1: dosage-sensitive sex reversal adrenal hypoplasia congenita critical region on the X chromosome: gene 1; DBD: DNA-binding domain; 6-ECDCA, 6-ethylchenodeoxycholic acid; EID1: E1A-like inhibitor of differentiation 1; ER: estrogen receptor; ERR*γ*: estrogen receptor-related receptor-*γ*; FBP1: fructose-1,6-bisphosphatase; FXR: farnesoid X receptor; G6Pase: glucose-6-phosphase; GR: glucocorticoid receptor; GPS2: G protein pathway suppressor 2; HD: enoyl-CoA hydratase/3-hydroxyacyl-CoA dehydrogenase; HDACs: histone deacetylases; HDAC-1: histone deacetylase-1; HDAC-1: histone deacetylase-3; JunD: predominat Jun family protein; HNF3/Foxa: hepatocyte nuclear factor-3; HNF4: hepatocyte nuclear factor-4; LPS: lipopolysaccharides; LXR*α*: liver X receptor*α*; LRH-1: liver receptor homologue-1; miRNAs (miR): microRNAs; NcoR: nuclear receptor corepressor; NF-*κ*B: nuclear factor-*κ*B; Nur77: nuclear growth factor I-B; PEPCK: phosphoenolpyruvate carboxykinase; PPRE: peroxisome proliferator-response elements; PXR: pregnane X receptors RAR: retinoid acid receptor; RNA Pol II: RNA polymerase II; RXR: retinoid X receptor; SIRT1: sirtuin1; SREBP-1c: sterol regulatory element-binding protein-1c; TCDD, 2,3,7,8-tetrachlorodibenzo-p-dioxin; TFIID: transcription initiation factor II D (TFIID); TFIIE: transcription factor II E; TGF-*β*: transforming growth factor-*β*; TLRs: Toll-like receptors; TR: thyroid receptor; TRAF6: TNF-receptor-associated factor-6; XRE, xenobiotic response element; YY1: Ying Yang 1.

**Table 4:** Studies on the association between *SHP (NR0B2) *genetic variation and birth weight, high BMI obesity, and fasting insulin diabetes.

Author	Country	Study populations/mutation	Subjects number	Mutation(s)	Association with birth weight increase	Association with BMI/ obesity	Association with increased insulin levels	Association with diabetes	Conclusions
Nishigori et al. [111]	Japan	Young-onset type 2 diabetes	274	In 7 subjects, 5 different mutations (H53fsdel10, L98fsdel9insAC, R34X, A195S, R213C) and 1 apparent polymorphism (R216H) (all in a heterozygous state)	Yes	Yes	—	No	*Shp* genetic variation: most common monogenic determinant of obesity and increased birth weight in Japanese

Hung et al. [110]	UK	GOOS (severe early-onset obesity)	329	R34G and R36C Missense mutations	Yes	No (selection of extreme obesity: stronger effect from other major gene?)	Yes	—	Genetic variation in the SHP locus may influence birth weight and have effects on BMI, possibly through effects on insulin secretion
			
				G171A (12%)	Yes		—	—	
		
				-195CTGAdel (16%) common polymorphisms	No (lower birth weight)		No (lower fasting levels)		
								
	UK	ALSPAC (cohort of children)	1,079	G171A	No	Yes (higher BMI and waist circumference at 7 yrs)	Yes (higher fasting levels and 30-min response)	—	Subtle effects in heterozygosity, stronger effects in homozygosity
			
				-195CTGAdel		No (lower BMI)		
							
	UK	Ely Study (Caucasian adults)	600	G171A	Data not available	Yes (BMI increased)	No	—
	
				-195CTGAdel		Yes (female: higher BMI), No (male: lower BMI)		

Mitchell et al. [113]	UK	Young-onset type 2 diabetes, obesity, birth weight	1,927	Birth weight: the only child homozygous for the A allele had a birth weight ≥4 kg	No	No	—	No	Mutations in SHP < UK than in Japanese obese type 2 subjects

									G171A coding polymorphism in 14.1% of UK subjects

									The A allele (G/A genotype) not associated with obesity or increased birth weight
				Obesity: no association if G/A genotype; yes (?) (if A/A homozygotes)		Yes (?)			Homozygous for the rare A allele: predisposed to moderate obesity and possibly increased birth weight

Echwald et al. [114]	Denmark	Early-onset obesity (men)	750	2 silent variants c.65C4T [p. Y22Y], c.339G4A [p. P113P]	—		—	—	Very low prevalence of functional SHP variants associated with obesity among Danes
		
				3 missense variants c.100C4G [p. R34G], c.278G4A [p. G93D], c.415C4A [p. P139H]		Yes (only among obese)			A role for G171A polymorphism low penetrance SHP variants) for obesity risk in Europe?
		
				G171A polymorphism (8.9%)		No (*P* = 0.07 versus obese)			Major differences in prevalence and impact of SHP variants between Danish and Japanese obese
		
		Nonobese controls	795	No variants G171A polymorphism (7.1)				
			
		Functional analyses in MIN6-m9 and HepG2 cell lines				93D mutant protein: reduced *in vitro *inhibition of the HNF4*α* transactivation of the HNF-1*α* promoter expression		

Note: SHP is expressed in the liver, pancreas, spleen, small intestine, and adrenal gland in humans [18] and inhibits the transcriptional activity of hepatocyte nuclear factor-4 *α* (HNF4*α*). ALSPAC: Avon Longitudinal Study of Parents and Children; GOOS: Genetics of Obesity Study; HNF4*α*: hepatocyte nuclear factor-4*α*.

## Abbreviations

AF1: Activation function 1
AF2: Activation function 2
AHR/ARNT: Aryl hydrocarbon receptor (AHR)/AHR nuclear translocator (ARNT)
ALDH3: Human aldehyde dehydrogenase 3 gene
AMPK: AMP-activated protein kinase
ARR: Adamantyl-substituted retinoid-related molecules
*β*1-AR: *β*1-adrenergic receptor
BAREs: Bile acids response elements
BAT: Brown adipose tissue
Brm: Human Brahma (a catalytic component of the SWI/SNF-related chromatin-remodeling complex)
CAR: Constitutive androstane receptor
CD437/AHPN: 6-[3-(1-Adamantyl)-4-hydroxyphenyl]-2-naphthalenecarboxylic acid
3-Cl-AHPC: 4-[3-(1-Adamantyl)-4-hydroxyphenyl]-3-chlorocinnamic acid
CYP3A: Hepatic cytochrome P-4503A
CYP1A1: Cytochrome P450, family 1, subfamily A, polypeptide 1
CYP1A2: Cytochrome P450, family 1, subfamily A, polypeptide 2
CYP1B1: Cytochrome P450, family 1, subfamily B, polypeptide 1
CYP2B1: Cytochrome P450, subfamily IIB
CYP7A1: Cholesterol 7 alpha-hydroxylase
CYP8B1: Cholesterol 12alpha-hydroxylase
DBD: DNA-binding domain
DAX-1: Dosage-sensitive sex reversal adrenal hypoplasia congenita (AHC) critical region on the X chromosome, gene 1
DR-4: Nuclear receptor half-site repeat
ER: Estrogen receptor
FBP1: Fructose-1,6-bisphosphatase
FGF: Fibroblast growth factor
FGFR: Fibroblast growth factor receptor
FISH: Fluorescence *in situ *hybridization
Foxo: Forkhead transcription factors
FXR: Farnesoid X receptor
G6Pase: Glucose-6-phosphatase
GPCR: G-protein-coupled Receptor
GPS2: G protein pathway suppressor 2
GR: Glucocorticoid receptors
HDL: High-density lipoprotein
HNF: Hepatocyte nuclear factor
JNK: Jun N-terminal kinase
JUN-D: Jun family of the activator protein 1 (AP-1) transcription factor complex
LBD: Ligand-binding domain
LDL: Low-density lipoprotein
LDLR: Low-density lipoprotein receptor
LRH1: Liver receptor homologue 1
3T3-L1: Mouse embryonic fibroblast adipose-like cell line.
LXXLL: Leu-Xaa-Xaa-Leu-Leu
mSin3A: Mammalian homolog of the Saccharomyces cerevisiae transcriptional corepressor Sin3p
N-CoR: Nuclear receptor corepressor
NRs: Nuclear receptors
PEPCK: Phosphoenolpyruvate carboxykinase
PGC-1: Peroxisome proliferator-activated receptor-*γ* (PPAR-*γ*) coactivator-1
PKC: Protein kinase C
PXR: Pregnane X receptor
RAR: Retinoid acid receptor
RNA Pol II: RNA polymerase II
RNF31: Member of the ring-between-ring (RBR) family of E3 ubiquitin ligases
RXR: Retinoid X receptor
SHP: Small heterodimer partner
SMILE: SHP-interacting leucine zipper protein
SMRT: Silencing mediator of retinoid and thyroid hormone receptor
SRC-1: Steroid receptor coactivator-1
SWI/SNF: SWItch/Sucrose NonFermentable (yeast nucleosome remodeling complex composed of several proteins which are products of the SWI and SNF genes)
UCP1: Uncoupling protein-1
USF-1: Upstream stimulatory factor-1
TFIID: Transcription initiation factor II D
TFIIE: Transcription factor II E
UGT-16: Uridine 5′-diphospho (UDP) glucuronosyl transferase family member
VLDL: Very-low-density lipoprotein
XREs: Xenobiotic response elements
WAT: White adipose tissue.
